# Construction and Evaluation of a Chimeric Japanese Encephalitis Virus Vaccine Candidate Strain with Chaoyang Virus as the Backbone

**DOI:** 10.3390/vaccines14010030

**Published:** 2025-12-26

**Authors:** Jiazhen Cui, Xuan Huang, Yupeng Li, Yuzhong Feng, Haolong Dong, Qingyang Wang, Xianghua Xiong, Xianzhu Xia, Gang Liu, Huipeng Chen

**Affiliations:** 1 Academy of Military Medical Sciences, Beijing 100850, China; 2 Institutes of Life Sciences and Medical Engineering, Anhui University, Hefei 230000, China

**Keywords:** flavivirus genus, insect-specific flavivirus, Chaoyang virus, chimeric vaccine, vaccine backbone, safety

## Abstract

Background: Pathogenic flaviviruses pose a serious threat to human health, and vaccines are an effective means of prevention and control. Although related vaccines have achieved significant progress, safety and efficacy limitations still exist, urgently requiring the development of novel vaccine platforms. The insect-specific flavivirus Chaoyang virus (CYV), with a structure similar to pathogenic flaviviruses and limited to insect cell replication, has potential as a safe vaccine vector. Methods: To systematically evaluate CYV’s potential as a universal flavivirus vaccine backbone and provide a vaccine candidate for type I Japanese encephalitis virus (JEV) prevention, this study constructed a chimeric JEV genotype I (GI) prME protein vaccine candidate CYV-JEV using CPER technology, systematically assessing its safety and immunoprotective effects. Results: Using the CPER method, CYV-JEV was successfully rescued, showing efficient replication in mosquito cells but defective replication in mammalian cells. As a vaccine backbone, CYV did not induce inflammatory responses or immune cell subset imbalances in IFNAR^−/−^ mice. CYV-JEV exhibited no pathogenicity in adult and suckling IFNAR^−/−^ mice. Immunisation of IFNAR^−/−^ mice with 10^6^ FFU twice provided complete protection against lethal challenge (100%) and effectively reduced paralysis rates (62.5%). Single-cell sequencing further revealed extensive T- and B-cell activation in the immune spleen. Conclusions: The results demonstrate that the CYV-based CYV-JEV candidate vaccine demonstrates both safety and efficacy, representing a promising alternative to attenuated JEV vaccines, with CYV showing potential as a safe and effective universal flavivirus vaccine backbone.

## 1. Introduction

Flavivirus is a group of enveloped, single-stranded positive-sense RNA viruses [1,2], with a genome length of approximately 11 kb [3]. The genome contains a single open reading frame encoding three structural proteins (capsid protein C, premembrane protein prM, and envelope protein E) and seven non-structural proteins (NS1, NS2a, NS2b, NS3, NS4a, NS4b, and NS5) [4]. Flaviviruses are classified according to their transmission vectors into mosquito-borne flaviviruses, transmitted between mosquitoes and vertebrates; tick-borne flaviviruses, transmitted between ticks and vertebrates; insect-specific flaviviruses, found only in mosquitoes and not infecting vertebrates; and viruses with undetermined transmission vectors [5,6]. Among mosquito-borne flaviviruses, several viruses pose serious threats to human health, including Dengue virus (DENV) [7], yellow fever virus (YFV), Zika virus (ZIKV) [8], West Nile virus (WNV) [9], and Japanese encephalitis virus (JEV) [10], which cause hundreds of millions of infections annually [11] and generate numerous severe cases and deaths, creating a substantial health burden globally [12,13,14].

Vaccines are an effective means of preventing pathogenic flaviviruses [15]; although pathogenic flaviviruses have caused a massive burden on global health, clinically approved vaccines are still lacking for most of them [16]. Flavivirus vaccine research strategies primarily include traditional live attenuated vaccines (such as CD.JEVAX^®^, SA14-14-2 strain) and inactivated vaccines (such as ENCEVAC^®^, Beijing-1 strain) [17], which have certain protective effects but still face challenges such as potential virulence reversion, high biosafety requirements, and limited immunoprotective effects [18]. In recent years, novel vaccine platforms represented by viral vector vaccines have gradually developed, such as Vesicular Stomatitis Virus (VSV), which primarily focuses on the key viral antigenic protein E [19], capable of efficiently inducing antibodies and providing protective effects, with short development cycles and high specificity, though the long-term safety of its backbone still requires in-depth evaluation. Urgent needs exist for safer and more effective vaccine solutions targeting pathogenic flaviviruses.

Insect-specific flaviviruses (ISFVs), which also belong to the genus Flavivirus, share similar structural characteristics with pathogenic flaviviruses [20]. As they can only replicate in insect vectors and are unable to infect vertebrate cells, they possess excellent biosafety properties and are ideal candidates for developing safe vaccine vectors [21]. Our team previously constructed a chimeric Zika virus vaccine candidate based on the Chaoyang virus, and preliminarily demonstrated its safety and efficacy at the animal level [22].

To further evaluate the safety and universality of the insect-specific flavivirus Chaoyang virus as a vaccine scaffold for pathogenic flaviviruses, we used Japanese encephalitis virus (JEV) as a model pathogen. Japanese encephalitis virus is the primary etiological agent of Asian viral encephalitis, severely threatening human health [23,24,25], and JEV vaccine research has yielded rich research outcomes and evaluation systems, such as the live attenuated vaccine SA 14-14-2, which played a crucial role in epidemic prevention and control [26]. We selected JEV as our research subject primarily based on two considerations: firstly, the existing live attenuated vaccine SA 14-14-2 is pathogenic and lethal in interferon-deficient mice [27], which can be used as an excellent evaluation platform whilst avoiding biological safety risks (achievable at BSL-2); secondly, the GI genotype of JEV is gradually replacing the GIII type as the predominant strain [28,29], and current vaccines have limited protective effects against the GI type, urgently necessitating a vaccine candidate specifically targeting GI.

Therefore, this study will graft the prME gene of GI-type JEV onto the CYV backbone, constructing a novel chimeric vaccine candidate strain (CYV-JEV), systematically evaluating its biological characteristics, backbone safety, chimeric vaccine candidate safety and efficacy, and further analysing its immunological features using single-cell sequencing technology. Through this research, we anticipate providing a safe and effective vaccine solution for GI-type JEV, whilst further validating the potential and value of insect-specific flavivirus CYV as a universal vaccine development platform.

## 2. Materials and Methods

### 2.1. Cells, Viruses, and Plasmids

Vero cells derived from African green monkey kidney (ATCC No.: CCL-81) and human embryonic kidney 293T cells (ATCC No.: CRL-3216) were maintained at 37 °C and 5% CO_2_ in a constant temperature incubator and cultured in DMEM medium; the medium was supplemented with 10% foetal bovine serum (FBS, Gibco, Grand Island, NE, USA), 10 mM HEPES buffer, and 1% penicillin-streptomycin mixture (P/S, Gibco, USA). White-striped mosquito C6/36 cells (ATCC No.: CRL-1660) were cultured at 28 °C, 5% CO_2_, using RPMI 1640 medium (provided by Gibco, USA), supplemented with 10% FBS, 10 mM HEPES, and 1% P/S to meet cell growth requirements. The Japanese encephalitis virus SA14-14-2 strain and Chaoyang virus (CYV) used in the experiment were constructed by virus rescue technology and subsequently preserved and passaged in this laboratory.

### 2.2. Viral RNA Extraction, RT-PCR Amplification, CPER, and Virus Rescue

The design of CYV-JEV involves replacing the prME gene of CYV (GenBank no.: NC_017086) with the prME gene of GI type JEV (GenBank no.: KT957422.1). To construct CYV-JEV, high-fidelity enzyme (Takara, Kyoto, Japan R026A) was used to amplify four DNA fragments using the CYV viral genome as a template via RT-PCR. The JEV prME fragment was synthesised from scratch using primers and assembled via PCA on an Oligo DX96 gene synthesiser. The linker fragment containing the mosquito cell OpIE2-CA promoter was previously designed and synthesised in the laboratory. After gel electrophoresis analysis, 6 amplified products were purified using a DNA purification kit. Amplification primers and recovered fragments are detailed in Supplementary Material S2.

CPER cyclisation reaction system: When configuring the reaction system, sequentially mix 6 equimolar (each 0.1 pmol) DNA fragments, 25 μL 2× Q5 high-fidelity premix enzyme, and an appropriate amount of ddH_2_O, ultimately bringing the total reaction volume to 50 μL. Subsequently, proceed with amplification under the following conditions: 98 °C initial denaturation for 30 s, followed by 12 cycling reactions, each cycle comprising 98 °C for 10 s, 55 °C for 20 s, and 68 °C for 6 min; finally, perform terminal extension at 68 °C for 10 min. After the reaction, take 5 μL of CPER product; according to the reagent instructions, add 2 μL Lipofectamine 3000 and 2 μL P3000 transfection reagent, which are directly used for transfecting C6/36 cells (6-well plate culture) with approximately 80% confluence. On the 6th day post-transfection, transfer cell supernatant to newly prepared C6/36 cells and continuously cultivate until a cytopathic effect appears, at which point collect cell supernatant for subsequent use.

### 2.3. Immunofluorescence Assay of CYV-JEV E Protein Expression

Vero, 293T, RIG-I^−/−^ 293T, MDA5^−/−^ 293T, and C6/36 cells grown in 24-well plates and confluent were inoculated with CYV-JEV at an MOI of 1 and incubated at 37 °C or 28 °C, respectively. After 3 days, cells were fixed and permeabilised with pre-cooled acetone-methanol (1:1) mixture for 10 min. Subsequently, cells were blocked with 1% BSA (in PBS) at 37 °C for 1 h. After blocking, cells were incubated with mouse monoclonal anti-yellow fever virus envelope protein antibody (1:200 dilution) at 37 °C for 1 h. After washing three times with PBS, cells were incubated with Alexa Fluor 488-labelled goat anti-mouse IgG secondary antibody (1:200 dilution) at 37 °C for 1 h. Finally, cell nuclei were stained with DAPI for 10 min. All fluorescence images were captured using a Zeiss Axio Observer inverted fluorescence microscope.

### 2.4. Cell Pathological Effect Analysis and Microscopic Observation

Detach the confluent C6/36 cells grown in 6-well plates and inoculate with CYV-JEV at an MOI of 1. After adsorption for 1 h at 5% CO_2_ and 28 °C, gently wash the cells once with PBS, and replace with RPMI 1640 maintenance medium containing 2% foetal bovine serum, 10 mM HEPES, and 1% penicillin-streptomycin. Continue incubation at 28 °C with 5% CO_2_. Observe and record the occurrence of cytopathic effects daily using a Zeiss Axio Observer inverted fluorescence microscope, and capture images simultaneously.

### 2.5. CYV-JEV Growth Kinetics

Inoculate C6/36 cells grown to approximately 80% confluence in 12-well plates with CYV-JEV at an MOI of 0.1, adsorb for 2 h, then wash thrice with PBS, subsequently maintain culture in DMEM medium containing 2% FBS, 10 mM HEPES, and 1% penicillin-streptomycin at 28 °C, 5% CO_2_. Continuously culture for 5 days, collecting 20 μL of cell culture supernatant daily.

Using a QuantStudio 3 real-time fluorescent quantitative PCR system, M5 HiPer Direct Viral RNA qPCR kit, specific TaqMan probes and corresponding primers, viral RNA in the harvested supernatant was quantitatively analysed. The total volume of the real-time fluorescent quantitative PCR reaction was 20 µL, comprising 14 µL buffer, 1 µL (10 µM) forward and reverse primers, 1 µL (10 µM) probe, 1 µL enzyme mixture, and 2 µL viral RNA template. The reaction programme was set as follows: pre-denaturation at 50 °C for 10 min; denaturation at 95 °C for 3 min; followed by 40 cycles of 95 °C for 10 s, 60 °C for 30 s (fluorescence signal collected at this stage). Viral RNA quantification was completed using the standard curve method.

### 2.6. Animal Experiments

All animal experimental procedures were strictly performed in accordance with the relevant regulations of the Chinese ‘Regulations on the Management of Experimental Animals’ and ‘Requirements for Experimental Animal Environment and Facilities’, and the overall experimental protocol was approved by the Experimental Animal Ethics Committee of the Military Medical Sciences Academy (Approval Number: IACUC-DWZX-2025-P026). The IFNAR gene knockout C57BL/6 mice used in this study were of a specific pathogen-free (SPF) strain, purchased from Beijing Huafu Kang Biotechnology Co., Ltd., Beijing, China, and housed in a standardised manner within the animal centre barrier system. During the experiment, mice in each inoculation group were co-housed in individual rodent cages, and all procedures were conducted in a Biosafety Level 2 (BSL-2) laboratory environment.

#### 2.6.1. Experiment I: Safety Evaluation of CYV Backbone in IFNAR^−/−^ C57BL/6 Adult Mice

Six-week-old SPF-grade IFNAR^−/−^ C57BL/6 mice were randomly divided into 2 groups. One group of mice were subcutaneously inoculated with 10^6^ FFU of CYV strain (*n* = 8), whilst the other group were subcutaneously inoculated with an equivalent volume of PBS control (*n* = 8). Body weight changes, clinical symptoms, and mortality were observed and recorded daily for 28 consecutive days. Peripheral blood samples of approximately 20 μL were collected via tail vein at 1, 3, 5, and 7 days post-inoculation, and multiple inflammatory markers were detected using a multiplex detection kit (LEGENDplex™ Mouse Inflammation Panel, BioLegend, San Diego, CA, USA) according to the manufacturer’s instructions. Mice were euthanised on day 14, and spleen and peripheral blood were collected to analyse immune cell subpopulations and proportions using multicolour flow cytometry (gating strategy for flow cytometry subsets detailed in Figures S1 and S2).

#### 2.6.2. Experiment II: Safety Evaluation of CYV-JEV in IFNAR^−/−^ C57BL/6 Adult Mice

Six-week-old SPF-grade IFNAR^−/−^ C57BL/6 mice were randomly divided into 2 groups (*n* = 5). Mice in both groups were intraperitoneally inoculated with 10^6^ FFU of CYV-JEV strain and JEV SA 14-14-2 (abbreviated as JEV-SA). Body weight changes and mortality were monitored daily for 14 consecutive days. Mice inoculated with JEV died on the 7th or 8th day post-infection, and brain, spleen, liver, and kidney tissues were collected. Mice inoculated with JEV were euthanised after the 14-day monitoring period, and corresponding organs were collected. Viral nucleic acid content in tissues and organs was detected, and tissue HE staining was performed.

#### 2.6.3. Experiment III: CYV-JEV Safety Evaluation in IFNAR^−/−^ C57BL/6 Suckling Mice Model

Three-day-old SPF-grade IFNAR^−/−^ C57BL/6 suckling mice were randomly divided into two groups (*n* = 8). Both groups of neonatal mice were subcutaneously inoculated with 20 FFU of JEV-SA and CYV-JEV. Body weight changes and mortality were monitored daily for 10 consecutive days. Neonatal mice inoculated with JEV-SA died on the 4th day of infection, and their brain, spleen, liver, and kidney tissues were collected. Neonatal mice inoculated with CYV-JEV were euthanised after the 10-day monitoring period, and corresponding organs were collected to detect viral nucleic acid content.

#### 2.6.4. Experiment IV: Immunogenicity Evaluation of CYV-JEV in IFNAR^−/−^ C57BL/6 Mice

Six-week-old SPF-grade IFNAR^−/−^ C57BL/6 mice were randomly divided into experimental and control groups. Mice in the experimental group were intraperitoneally inoculated with CYV-JEV at 10^4^/10^5^/10^6^ FFU, either once or twice (with a 14-day interval) (*n* = 8), whilst control group mice were intraperitoneally inoculated with the same volume of PBS at the same frequency (*n* = 6). After 4 weeks of immunisation, mice were challenged with 10^5^ FFU of JEV-SA. Body weight, survival rate, and clinical signs (paralysis rate) were monitored continuously for 14 days post-challenge. Blood was collected via tail snip on the 6th day post-challenge to detect viraemia; mice were euthanised either upon death or after the 14-day observation period, with brain tissue collected to detect viral nucleic acid.

### 2.7. Viral Genome Detection by qRT-PCR

Homogenise various tissues and organs using a grinding apparatus. According to the manufacturer’s instructions, perform absolute quantification of viral RNA in blood samples and organ tissue homogenates using the M5 HiPer Direct Viral RNA qPCR Kit. Conduct TaqMan one-step quantitative reverse transcription PCR reactions on a real-time fluorescence quantitative PCR system, utilising specific primers and probes targeting the JEV-SA NS3 gene (forward primer: 5′-AGAGCACCAAGGGAATGAAATAGT-3′; probe: 5′-(FAM) CCACGCCACTCGACCCATAGACTG (BHQ1)-3′; reverse primer: 5′-AATAGGTTGTAGTTGGGCACTCTG-3′), and apply the standard cycling programme. Calculate viral load based on a standard curve prepared from 10-fold diluted viral RNA, with final results expressed as the logarithm of viral RNA copy numbers per gramme of tissue or per millilitre of blood.

### 2.8. Antibody Detection

#### 2.8.1. Experiment I: Neutralising Antibody Detection

We used plaque reduction neutralisation test to detect JEV-specific neutralising antibodies in serum, with the following specific steps: Inactivated serum was serially diluted in a 96-well plate (initial dilution of 1:10, twofold gradient dilution to 1:1280), using virus maintenance medium without serum (DMEM + 2% FBS) as the diluent. Four replicates were set for each dilution. JEV-SA virus stock was diluted to working concentration (approximately 100–200 FFU/50 μL). Equal volumes of diluted serum and virus working solution were mixed, gently blown and mixed, then incubated at 37 °C and 5% CO_2_ for one hour to allow neutralising antibodies to bind with the virus. Vero E6 cells (cultured in 96-well plates) with confluent monolayers were gently washed once with PBS, then the PBS was discarded. Virus-serum mixture was added to cell wells and continuously cultured at 37 °C for three days until obvious CPE appeared in positive control wells. Neutralising antibody titre was calculated by observing cell pathogenic effects (CPE) at various serum dilutions. The Reed–Muench method was used to calculate neutralising antibody levels, with serum dilution reciprocal inhibiting 50% plaque formation or CPE defined as half neutralisation titre. Serum 50% neutralisation titre was calculated using linear interpolation via Prism 8.0.2 software.

#### 2.8.2. Experiment II: Detection of JEV IgG Antibody Levels and nAbs Levels

The JEV IgG antibody levels and nAbs titres in serum were detected using a commercial mouse JEV-IgG enzyme-linked immunosorbent assay kit (MEIMIAN, MM-50213O1, MM-44857M1). Brief procedure: Dilute test serum samples 5-fold and add to enzyme-labelled plate, simultaneously setting standard curve wells and blank control wells. Incubate at 37 °C for 30 min, wash plate, and add horseradish peroxidase-labelled detection antibody for continued incubation. After further washing, sequentially add colour reagents A and B for colour development in dark conditions, then add termination solution to stop the reaction. Immediately measure the optical density of each well at 450 nm wavelength using an enzyme-labelled instrument. Construct a standard curve based on standard concentration and OD values, and calculate the relative concentration of JEV antibodies in the test samples.

### 2.9. Single-Cell Sequencing Sample Preparation

To elucidate the mechanism of immune response, mice were sacrificed on day 28 post-immunisation, and the spleen was aseptically harvested. The spleen was homogenised in pre-chilled PBS through a 70 μm cell strainer to prepare a single-cell suspension. Following red blood cell lysis using ACK lysing solution, cells were washed and resuspended, with cell viability >85% confirmed by trypan blue staining. Subsequently, using the 10x Genomics platform and strictly adhering to the operation manual, single-cell sorting, barcode labelling, and cDNA library construction were performed. The final library was sequenced on the sequencing platform.

### 2.10. Data Analysis

All data analyses in this study were completed using GraphPad Prism 8.0.2 software. Bodyweight comparisons between groups were performed using two-way analysis of variance. Survival rate analysis was conducted using the Kaplan–Meier method to plot survival curves, with inter-group differences compared using log-rank tests. For viral load, virus titres and viral RNA levels were log-transformed prior to statistical analysis using unpaired *t*-tests. *p*-values of less than 0.05 were considered statistically significant.

## 3. Results

### 3.1. Rescuing Chimeric Virus CYV-JEV Using CPER Technology in Mosquito Cells

**Figure 1:**
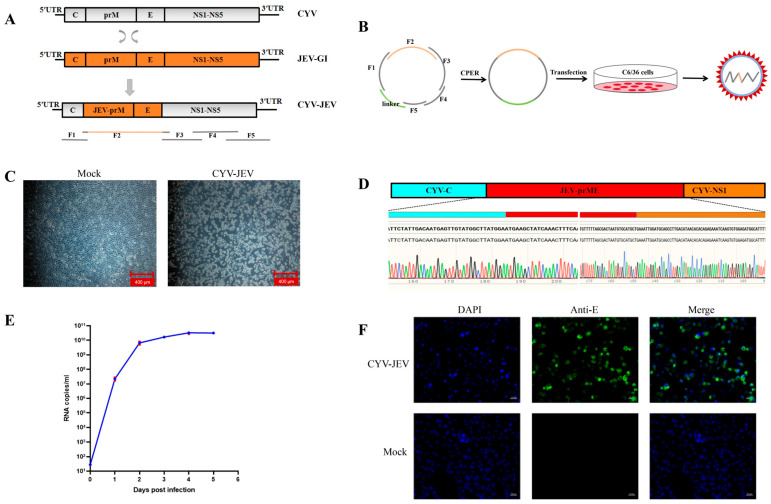
Construction and verification of chimeric virus CYV-JEV. (**A**): Schematic diagram of chimeric virus construction strategy. (**B**): CPER reaction cyclisation and assembly of six DNA fragments. (**C**): Mock group as negative control cells; CYV-JEV group showing obvious cytopathic effect, necrosis, and cell detachment after 5 days of infection. (**D**): Sanger sequencing of chimeric virus genome; results showing the prME region sequence of rescued CYV-JEV is virus completely identical to JEV target sequence. (**E**): One-step growth curve of chimeric virus, virus titre reaching peak on day 4 of C6/36 cell infection. (**F**): Indirect immunofluorescence results; CYV-JEV group capable of normal and effective JEV E protein expression.

The main construction strategy of chimeric virus CYV-JEV was to replace the prME region of Chaoyang virus with the corresponding region of JEV (Figure 1A). First, the backbone genome fragments were amplified in four segments using RT-PCR with Chaoyang virus genome as the template, and the prME fragment of Type I JEV (GenBank accession: KT957422.1) was assembled through PCA splicing and yeast homologous recombination, with each fragment containing a homologous arm for subsequent circular assembly. The chimeric virus genome sequence is shown in Supplementary Material S1. A linker fragment containing the insect-specific promoter OpIE2-CA was used to achieve circularisation [30]. Six DNA fragments were circularly assembled via CPER reaction (Figure 1B). The CPER product was transfected into C6/36 cells, and at 6 days post-transfection, the cell supernatant was collected and inoculated onto fresh C6/36 cells, with an obvious cytopathic effect observed at 5 days post-inoculation, indicating successful virus rescue (Figure 1C). Genome sequencing by the Sanger method verified that the prME region sequence of the rescued CYV-JEV virus was identical to the JEV target sequence, with the junction and flanking sequences matching the expected design (Figure 1D). To further evaluate viral replication capacity, the virus supernatant was inoculated into C6/36 cells, and viral copy numbers were collected and detected for five consecutive days to plot a one-step growth curve, which demonstrated that the virus exhibited excellent replication efficiency in insect cells (Figure 1E). Indirect immunofluorescence results further confirmed that CYV-JEV-infected cells effectively expressed JEV E protein and could be recognised by flavivirus E protein-specific monoclonal antibodies (Figure 1F). In summary, this study successfully rescued a chimeric virus, CYV-JEV, with strong replication capacity and correct antigen expression using the CPER method in C6/36 cells.

### 3.2. Evaluation of Replication Characteristics of Chimeric Virus CYV-JEV in Vertebrate Cells

**Figure 2:**
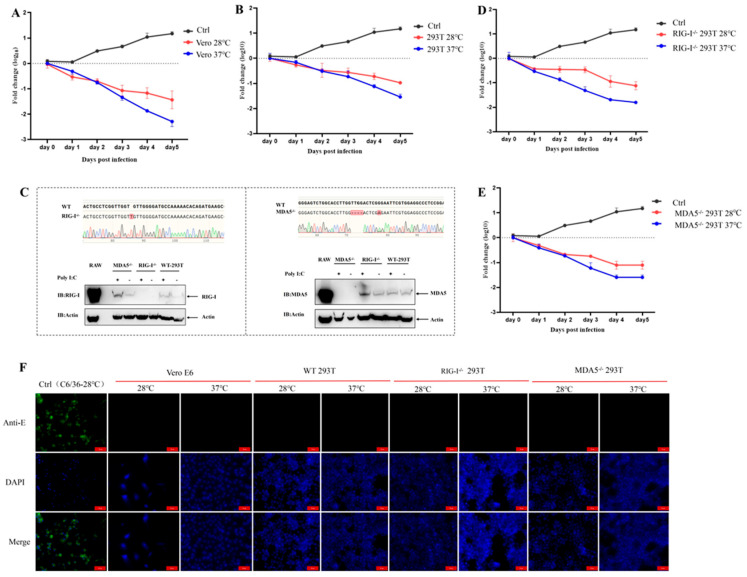
Chimeric virus CYV-JEV does not infect vertebrate cells. (**A**): Chimeric virus CYV-JEV viral nucleic acid copy numbers over infection time at 28 °C and 37 °C in Vero E6 cells. (**B**): Chimeric virus CYV-JEV viral nucleic acid copy numbers over infection time at 28 °C and 37 °C in 293T cells. (**C**): CRISPR/Cas9 technology used to construct RIG-I^−/−^ 293T and MDA5^−/−^ 293T cells, with Sanger sequencing and Western blot verifying successful immunodeficient cell construction. (**D**): Chimeric virus CYV-JEV viral nucleic acid copy numbers over infection time at 28 °C and 37 °C in RIG-I^−/−^ 293T cells. (**E**): Chimeric virus CYV-JEV viral nucleic acid copy numbers over infection time at 28 °C and 37 °C in MDA5^−/−^ 293T cells. (**F**): Indirect immunofluorescence verifying CYV-JEV unable to express JEV E protein in Vero E6, 293T, RIG-I^−/−^ 293T, and MDA5^−/−^ 293T cells under 37 °C and 28 °C culture conditions.

To evaluate whether the chimeric virus CYV-JEV retained the non-infectivity of its parental Chaoyang virus towards vertebrate cells, we first detected its replication capacity in two standard vertebrate cell lines: Vero E6 (African green monkey kidney cells) and HEK 293T (human embryonic kidney cells). To investigate the effect of culture temperature on viral replication capacity simultaneously, we cultured virus-infected cells at 37 °C and 28 °C, respectively. Viral supernatant was inoculated into corresponding cells, and nucleic acid levels of infected cells were monitored continuously for 5 days. Using infection days as the abscissa and logarithm of copy numbers relative to day 0 as the ordinate, a one-step growth curve was plotted using GraphPad Prism 8.0.2. Positive values indicate viral nucleic acid content increase in cell culture supernatant, representing effective viral replication and amplification; negative values indicate viral nucleic acid reduction, suggesting ineffective viral replication and gradual degradation of supernatant nucleic acid content. Results showed that after inoculating CYV-JEV into Vero E6 and 293T cells, viral nucleic acid levels continuously decreased relative to day 0 of infection, with no effective amplification observed (Figure 2A,B). We further detected the expression of CYV-JEV-specific E protein antigen using indirect immunofluorescence. Results demonstrated no E protein expression in infected Vero and 293T cells (Figure 2F). These data indicate that CYV-JEV cannot effectively infect or replicate in Vero E6 and 293T cells.

Considering that the replication of mosquito-borne viruses in mammalian cells might be restricted by the innate immune system, we further investigated the roles of key viral pattern recognition receptors RIG-I and MDA5 in CYV-JEV infection limitation. To this end, we successfully constructed RIG-I knockout (RIG-I^−/−^) and MDA5 knockout (MDA5^−/−^) 293T cell monoclonal deficient strains using CRISPR/Cas9 technology (Figure 2C). In RIG-I^−/−^ 293T and MDA5^−/−^ 293T cells, we repeated the CYV-JEV infection experiment. The results showed that even in RIG-I^−/−^ 293T and MDA5^−/−^ 293T cells, CYV-JEV remained unable to achieve effective amplification during passaging (Figure 2D,E). Corresponding immunofluorescence detection also confirmed that CYV-JEV E antigen protein was negative in these immunodeficient cells (Figure 2F). These data indicate that CYV-JEV cannot effectively replicate in vertebrate cells, and this replication characteristic is not limited by viral pattern recognition receptors RIG-I and MDA5.

### 3.3. Safety Evaluation of the CYV Backbone in Murine Models

**Figure 3:**
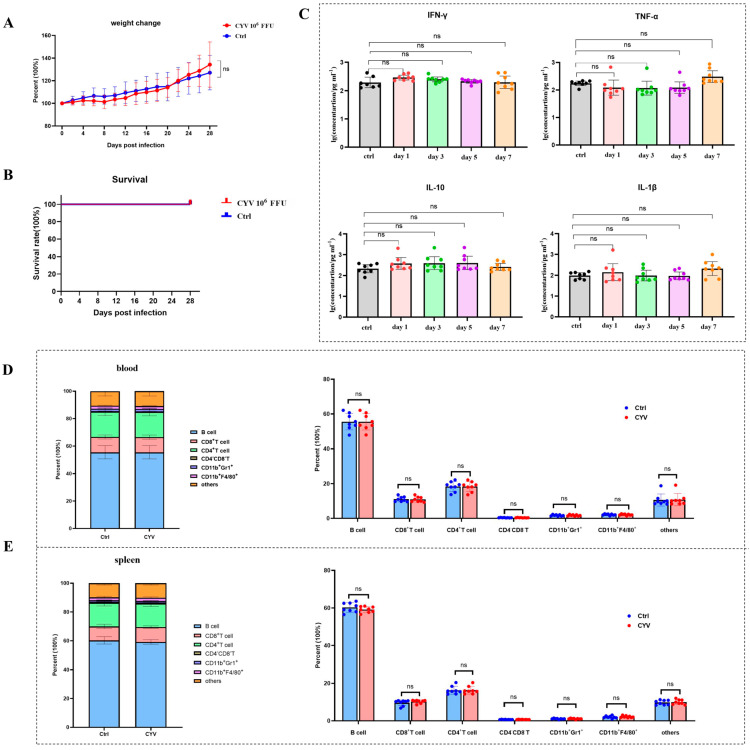
Safety evaluation of CYV as a vaccine scaffold. (**A**): Continuous monitoring of body weight changes in mice after CYV inoculation for 28 days, with no significant differences compared to the control group. (**B**): Continuous monitoring of survival rates in mice after CYV inoculation for 28 days, with all mice surviving during the monitoring period and no significant differences compared to the control group. (**C**): Detection of peripheral blood inflammatory factors IFN-γ, TNF-α, IL-27, and IL-1β in mice at days 1, 3, 5, and 7 after CYV inoculation, with results showing no significant increases or abnormal fluctuations at all detection time points. (**D**): Multicolour flow cytometry analysis showing no abnormal changes in peripheral blood immune cell subsets and proportions on day 14 after CYV inoculation. (**E**): Multicolour flow cytometry analysis showing no abnormal changes in splenic immune cell subsets and proportions on day 14 after CYV inoculation. ns *p* > 0.05.

To assess the safety of Chaoyang virus (CYV) as a vaccine scaffold, we selected an immunodeficient (IFNAR^−/−^) C57BL/6 mouse model that is sensitive to flaviviruses for safety analysis. Six-week-old adult mice were intraperitoneally inoculated with 10^6^ FFU of CYV (*n* = 8) and observed continuously for 28 days, monitoring body weight changes and survival, with the control group receiving an equivalent volume of PBS (*n* = 8). Results showed that compared to the control group, CYV-inoculated mice did not experience mortality throughout the observation period, and body weight change trends were essentially consistent, with no significant differences (Figure 3A,B), indicating that the CYV was non-lethal to IFNAR^−/−^ C57BL/6 mice and did not cause apparent systemic adverse reactions. To further investigate whether CYV induced severe inflammatory responses in mice, we collected mouse sera on days 1, 3, 5, and 7 post-inoculation and analysed the dynamic changes in key inflammatory factors (IFN-γ, TNF-α, IL-27, and IL-1β) using a multi-factor detection kit. Data revealed that, compared to the control group, the aforementioned cytokine levels in the experimental group did not exhibit significant increases or abnormal fluctuations at all detected time points (Figure 3C), suggesting that CYV inoculation did not provoke intense immune inflammatory responses. Mice were sacrificed on day 14, and the distribution of major immune cell subsets in peripheral blood and spleen was analysed by multicolour flow cytometry to further evaluate the impact of CYV on the immune system at the cellular level. Results showed no statistically significant differences in the proportions of key immune cells, including T cells, B cells, and macrophages, between the experimental and control groups in peripheral blood and spleen (Figure 3D,E), with flow cytometry gating strategies detailed in Figures S1 and S2. Taken together, these in vivo experimental data demonstrate that in the IFNAR^−/−^ C57BL/6 mouse model, CYV exhibits good safety, without causing significant pathological reactions, inflammatory responses, or immune cell proportion imbalances.

### 3.4. Safety Evaluation of CYV-JEV Chimeric Virus in IFNAR^−/−^ C57BL/6 Adult Murine Model

**Figure 4:**
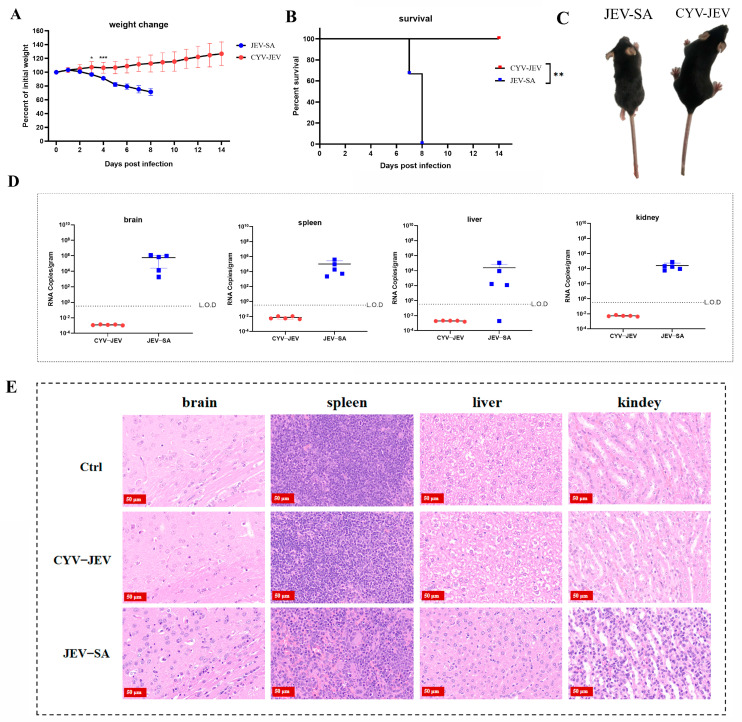
Safety evaluation of chimeric virus CYV-JEV in adult mice. (**A**): Monitoring of body weight changes in mice intraperitoneally inoculated with CYV-JEV or JEV-SA over 14 consecutive days. (**B**): Monitoring of survival rate changes in mice intraperitoneally inoculated with CYV-JEV or JEV-SA over 14 consecutive days. (**C**): Morphological signs of mice on day 7 after intraperitoneal inoculation with CYV-JEV or JEV-SA, with JEV-SA group mice showing hind limb paralysis, ruffled fur, and lethargy and CYV-JEV group mice showing no abnormal clinical signs. (**D**): Viral nucleic acid content detected in brain tissue, spleen, liver, and kidney of mice from CYV-JEV and JEV-SA groups after death or sacrifice at the end of monitoring. (**E**): Histopathological analysis results of brain tissue, spleen, liver, and kidney tissues from CYV-JEV, JEV-SA, and Ctrl groups. * *p* ≤ 0.05, ** *p* ≤ 0.01,*** *p* ≤ 0.001.

To evaluate the safety of chimeric virus CYV-JEV in adult mice, we similarly selected the IFNAR^−/−^ C57BL/6 mouse model susceptible to flaviviruses, using JEV SA14-14-2 (abbreviated as JEV-SA) as a positive control for safety assessment. After intraperitoneal inoculation of 10^6^ FFU of the respective viruses (CYV-JEV or JEV-SA), mice were systematically monitored for body weight changes, survival rate, and clinical signs for 14 consecutive days. Experimental results showed that mice in the JEV-SA inoculation group began progressive weight loss from the second day of infection, started dying from the seventh day, and were completely dead by the eighth day (Figure 4A,B). JEV-SA-infected mice exhibited typical neurological symptoms, including arched back, ruffled fur, lethargy, and limb paralysis (Figure 4C and Figure S3). Viral nucleic acid was detected at high levels in the spleen, liver, kidney, and brain tissues of deceased mice, indicating viral invasion of critical organs (Figure 4D). All mice in the CYV-JEV inoculation group survived, with body weight steadily increasing by approximately 30%, and no abnormal clinical symptoms were observed. Viral nucleic acid was not detected in the spleen, liver, kidney, and brain tissues of mice euthanised on the 14th day post-inoculation (Figure 4D). Histopathological analysis showed that JEV-SA-inoculated mice exhibited significant lesions in the spleen, brain, liver, and kidney tissues, including cell necrosis and inflammatory cell infiltration, whereas the CYV-JEV-inoculated and negative control groups displayed normal tissue structures with no apparent inflammatory responses (Figure 4E). These results indicate that CYV-JEV demonstrates good safety in the IFNAR^−/−^ C57BL/6 adult mouse model.

### 3.5. Safety Evaluation of CYV-JEV Chimeric Virus in IFNAR^−/−^ C57BL/6 Suckling Mice Model

**Figure 5:**
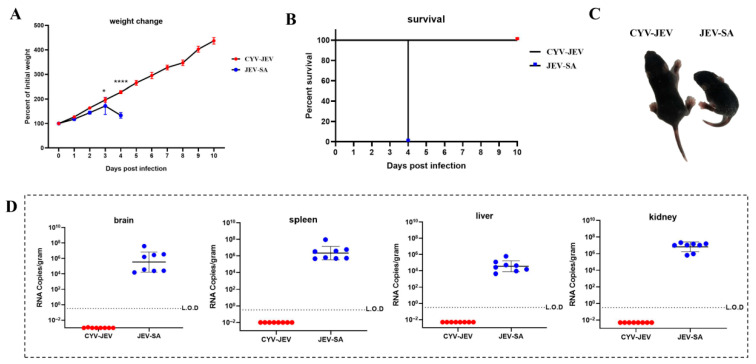
Safety evaluation of chimeric virus CYV-JEV in suckling mice. (**A**): Body weight changes in suckling mice continuously monitored for 10 days after subcutaneous inoculation with CYV-JEV or JEV-SA. (**B**): Survival rate changes in suckling mice continuously monitored for 10 days after subcutaneous inoculation with CYV-JEV or JEV-SA. (**C**): Morphological signs of suckling mice on the 4th day after subcutaneous inoculation with CYV-JEV or JEV-SA, with JEV-SA group mice appearing lethargic and smaller and CYV-JEV group mice showing no abnormal clinical signs. (**D**): Viral nucleic acid content in brain tissue, spleen, liver, and kidneys of suckling mice in the CYV-JEV and JEV-SA groups after death or sacrifice at the end of monitoring. * *p* < 0.05, **** *p* < 0.0001.

We further adopted the IFNAR^−/−^ C57BL/6 neonatal mouse model with higher susceptibility to Japanese encephalitis virus for safety verification. Three-day-old IFNAR^−/−^ C57BL/6 mice were selected and subcutaneously inoculated with 20 FFU of JEV-SA or CYV-JEV, with systematic monitoring of mouse body weight changes, survival rate, and clinical signs for 10 consecutive days. Results showed that the JEV-SA-inoculated mice experienced significant body weight decline from day 4 and died completely (Figure 5A,B), with JEV-SA-inoculated mice being notably smaller in body size compared to CYV-JEV-inoculated mice (Figure 5C); high viral titres were detected in liver, kidney, spleen, and brain tissues, indicating viral invasion of critical organs (Figure 5D). In contrast, CYV-JEV-inoculated mice exhibited a four-fold body weight increase during the 10-day observation period, with no infection signs or mortality, and no viral nucleic acid detected in key tissues (Figure 5D). These results strongly demonstrate that CYV-JEV maintains excellent safety even in an immunodeficient neonatal mouse model.

### 3.6. Immunogenicity and Protective Efficacy Evaluation of CYV-JEV

To evaluate the immunogenicity and protective efficacy of the CYV-JEV candidate vaccine, this study used six-week-old IFNAR^−/−^ C57BL/6 mice as a model, randomly dividing the mice into experimental and control groups. The experimental group received intraperitoneal injections of different doses of CYV-JEV (*n* = 8), whilst the control group received equivalent volumes of PBS (*n* = 6). The experiment established three dose gradients (10^4^, 10^5^, and 10^6^ FFU), with single and two-dose immunisation protocols. Serum was collected 28 days post-immunisation to detect neutralising antibody levels, JEV-specific antibodies (JEV-nAb), and IgG antibody levels. At 30 days post-immunisation, mice were challenged with 10^5^ FFU JEV-SA, with systematic monitoring of body weight changes, clinical manifestations, survival rate, and paralysis incidence. Viral nucleic acid loads in blood and brain tissues were detected using qRT-PCR.

**Figure 6:**
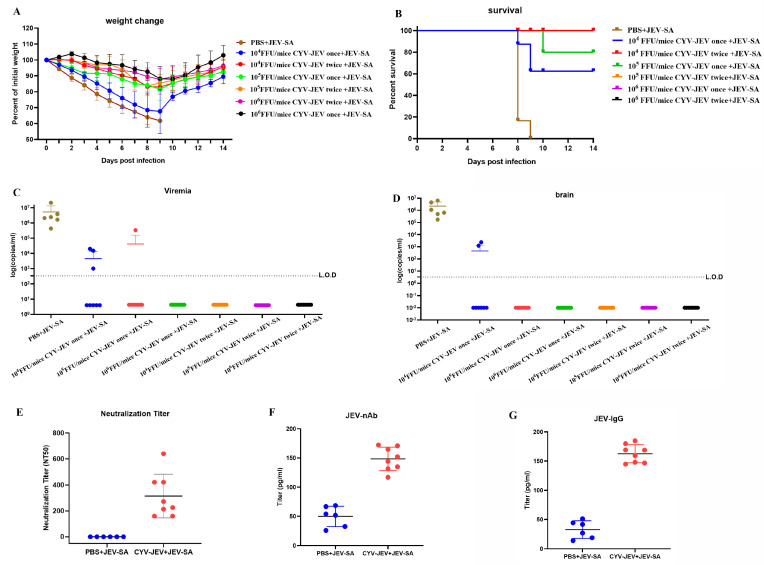
Efficacy and protective effect evaluation of chimeric virus CYV-JEV. (**A**): Mice intraperitoneally inoculated with different doses and frequencies of CYV-JEV and challenged with JEV-SA, with body weight changes monitored continuously for 14 days. (**B**): Mice intraperitoneally inoculated with different doses and frequencies of CYV-JEV and challenged with JEV-SA, with survival rate changes monitored continuously for 14 days. (**C**): Peripheral blood viral nucleic acid copy number detection on day 6 in mice intraperitoneally inoculated with different doses and frequencies of CYV-JEV and challenged with JEV-SA. (**D**): Mice intraperitoneally inoculated with different doses and frequencies of CYV-JEV and challenged with JEV-SA, viral nucleic acid content detected in brain tissue after death or end of monitoring period. (**E**): Serum neutralising antibody titre (NT50) detection on day 28 with intraperitoneal inoculation of 10^6^ FFU CYV-JEV with two immunisation doses. (**F**): ELISA detection of serum JEV-nAb on day 28 with intraperitoneal inoculation of 10^6^ FFU CYV-JEV with two immunisation doses. (**G**): ELISA detection of serum JEV-IgG on day 28 with intraperitoneal inoculation of 10^6^ FFU CYV-JEV with two immunisation doses.

Results showed that all CYV-JEV-immunised groups experienced temporary body weight reduction following viral challenge, which gradually recovered (Figure 6A). In the single immunisation group, protective efficacy significantly increased with higher immunisation doses: the 10^4^ FFU group had a survival rate of 62.5% (5/8), with all surviving mice exhibiting paralysis symptoms and occasional viral RNA detected in blood and brain tissues; the 10^5^ FFU group’s survival rate increased to 87.5% (7/8), with paralysis rate reduced to 62.5% and occasional viral nucleic acid positivity in blood; the 10^6^ FFU group achieved a 100% survival rate, with no viral nucleic acid detected in any tissue samples, although paralysis occurrence remained at 50%. In the two-immunisation protocol, survival rates for all dose groups reached 100%. Among these, the 10^4^ FFU and 10^5^ FFU groups both had a 62.5% paralysis rate with no JEV RNA detected in tissues; the 10^6^ FFU group’s paralysis rate further decreased to 37.5% (Figure S4), with no viral nucleic acid detected in blood or key tissues. Neutralising antibody, JEV-nAb, and JEV-IgG antibody levels were measured for mice in the 10^6^ FFU two-immunisation group, demonstrating that CYV-JEV immunisation effectively stimulates corresponding antibody production.

CYV-JEV demonstrated significant immunoprotective effects in the IFNAR^−/−^ mouse model, particularly with a two-dose immunisation regimen at 10^6^ FFU, achieving a 100% survival rate and a low paralysis rate (37.5%) and completely suppressing viral spread in blood and tissues.

### 3.7. Single-Cell Sequencing Reveals Immunological Response Characteristics Induced by CYV-JEV

In preliminary research, the chimeric Japanese encephalitis virus vaccine candidate strain (CYV-JEV) demonstrated good safety and immunoprotective efficacy in animal models. To further elucidate its immune mechanism, this study used splenic tissue from mice immunised with a single dose of 10^6^ FFU and examined 28 days post-vaccination (*n* = 6, pooled as one sample), conducting single-cell RNA sequencing (scRNA-seq) analysis. Utilising the 10× Genomics platform for library preparation and sequencing, the study performed raw data quality control, dimensionality reduction, clustering visualisation, differential expression analysis, cell subpopulation annotation, and pathway enrichment analysis.

**Figure 7:**
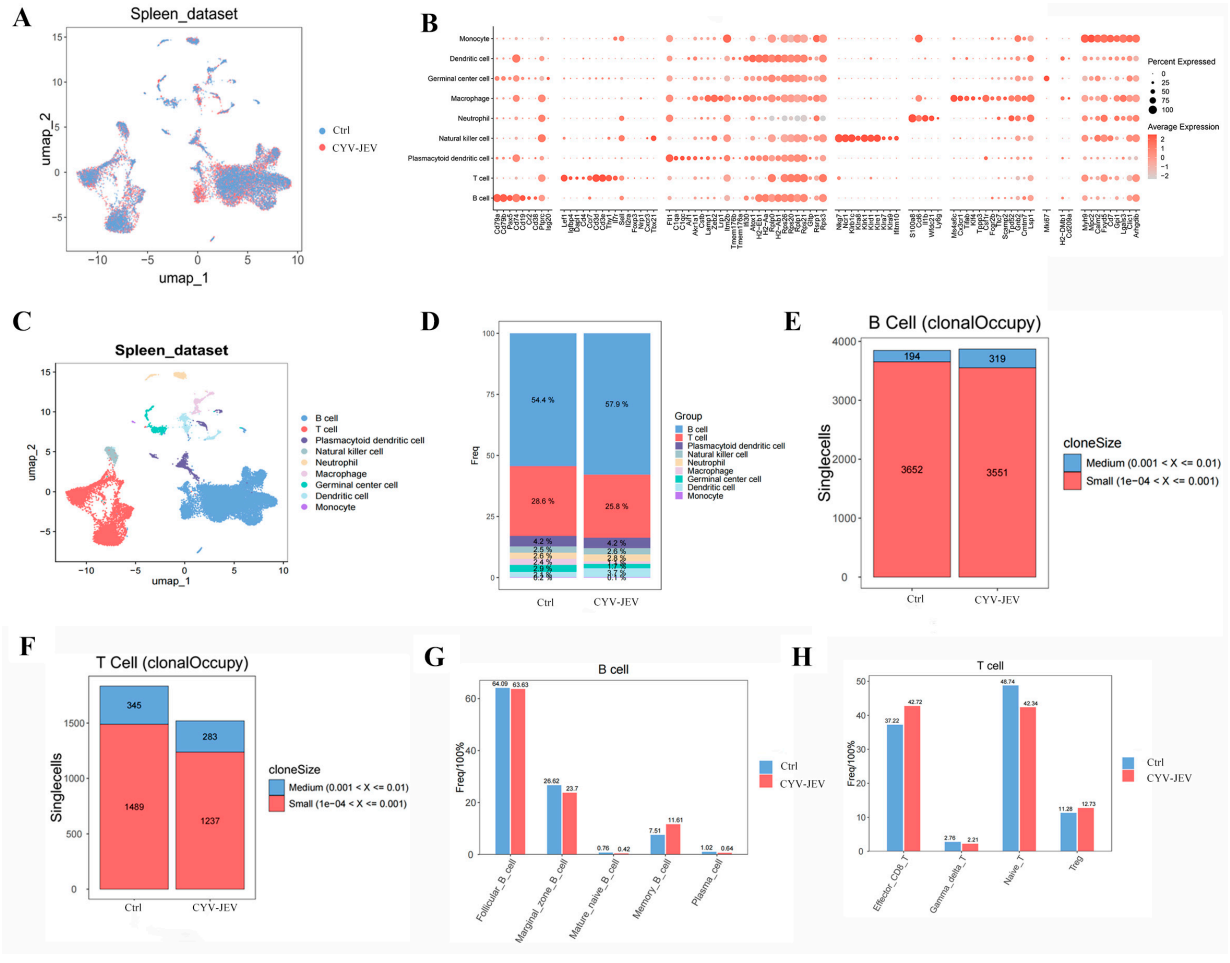
Single-cell sequencing analysis of immunological characteristics after CYV-JEV immunisation. (**A**–**C**): Visualisation of spleen cell clustering, differential expression analysis, and cell subtype annotation. (**D**): Statistical distribution of cell population proportions across different treatment groups. (**E**): B-cell receptor (BCR) analysis, proportion analysis of antibodies with different clone sizes. (**F**): T-cell receptor (TCR) analysis, proportion analysis of antibodies with different clone sizes. (**G**): Statistical distribution of B-cell subtype proportions. (**H**): Statistical distribution of T-cell subtype proportions.

Firstly, we identified and annotated the primary cell types based on specifically expressed marker genes in the splenic cell population, and calculated the proportion distribution of each cell population in different treatment groups (Figure 7A–D). The results showed that, compared with the control group, the total T-cell quantity decreased in the CYV-JEV-immunised group (from 28.6% to 25.8%), whilst the total B-cell quantity increased (from 54.4% to 57.9%). Further analysis of B-cell receptors (BCR) demonstrated that CYV-JEV immunisation prompted some B-cell populations originally belonging to the “small clone” category to proliferate to the “medium clone” category (small clone number reduced from 3652 to 3551, whereas medium clone number increased from 194 to 319), suggesting that CYV-JEV immunisation activated humoral immune responses (Figure 7E); in the T-cell receptor (TCR) analysis, the antibody content of different clone sizes was reduced (Figure 7F), indicating that CYV-JEV immunisation simultaneously mobilised T and B cells to participate in immune responses.

To deeply analyse the specific changes in these two cell types in immune response, we separately performed subgroup subdivision and functional analysis on B and T cells. B cells can be divided into five subgroups (Figure 7G), with population statistics showing a significant increase in Memory B-cell proportion in the CYV-JEV group (from 7.51% to 11.61%). Further analysis of antibody chain composition in this subgroup revealed a marked enhancement of three light chains originating from the IGK gene locus (see Figure S5). In T cells, we successfully annotated four subgroups (Figure 7F and Figure S6), where the proportions of both Effector CD8^+^ T and Treg in the CYV-JEV group significantly increased, with Effector CD8^+^ T rising from 37.22% to 42.72%, and Treg from 11.28% to 12.73%. Analysis of TCR composition in Effector CD8^+^ T cells showed a significant upregulation of four β chains related to the TRB gene in the CYV-JEV group (see Figure S6).

In summary, this study reveals, at the single-cell transcriptome level, the molecular basis of CYV-JEV simultaneously activating humoral and cellular immune responses: on the one hand, by promoting Memory B-cell proliferation and enhancing BCR diversity, strengthening the humoral immune response; on the other hand, by increasing the proportion of Effector CD8^+^ T cells and Treg and enriching specific TCR clones, synergistically enhancing the cellular immune response. These results indicate that the dual immune reactions induced by CYV-JEV jointly participate in its immune protective mechanism.

## 4. Discussion

Pathogenic flaviviruses, as important pathogens of emerging and re-emerging infectious diseases, have drawn global public health attention [31,32,33]. Although traditional vaccine development strategies have made certain progress, issues such as low vaccine safety, potential virulence reversion, and limited protective effects continue to constrain vaccine development. In recent years, innovative strategies based on insect-specific flaviviruses (ISFVs) have demonstrated unique advantages. This study successfully constructed a CYV-JEV candidate vaccine strain with chimeric JEV GI prME protein using CPER technology. In vitro and in vivo experimental results showed that CYV-JEV replicates efficiently in insect cells, while exhibiting replication defects in mammalian cells, fully embodying the core advantage of insect-specific flaviviruses as vaccine vectors—inherent biological safety. The CYV backbone and CYV-JEV chimeric virus exhibited excellent safety in IFNAR^−/−^ mice, which benefit from the inherent vertebrate replication defect of insect-specific flaviviruses—forming a ‘biophysical barrier’ that avoids pathogenicity risks associated with traditional attenuated vaccines. The 100% survival rate and reduced paralysis rate induced by two-dose 10^6^ FFU CYV-JEV, combined with single-cell sequencing evidence of T/B-cell activation, confirm that CYV-JEV elicits coordinated humoral and cellular immune responses. This dual immune activation not only explains its protective efficacy but also distinguishes it from vaccines relying solely on antibody induction, highlighting the advantage of the CYV platform in inducing comprehensive immunity. These results collectively demonstrate that the JEV candidate vaccine based on a CYV backbone demonstrates both safety and effectiveness.

The successful construction of CYV-JEV chimeric vaccine candidates highlights the immense potential and universal advantages of the ISFV platform represented by CYV in vaccine development. Firstly, there is its natural safety: its defective replication in vertebrates constitutes a “biophysical barrier”, greatly reducing virulence risks and offering superior safety compared to traditional vaccines. Secondly, there is its excellent immunogenicity: chimeric viruses can effectively present antigens, inducing comprehensive immune responses including neutralising antibodies and T-cell responses. Thirdly, there is its production convenience: it can proliferate efficiently in mosquito cells, reducing production costs and the requirement for high-level biosafety facilities. Lastly, there is its platform universality: this strategy can be extended to other flavivirus vaccine development merely by replacing the corresponding prME genes, enabling rapid vaccine development for emerging flaviviruses (such as West Nile virus and Zika virus) or new viral genotypes, providing a rapid response platform technology to address future viral mutations or emerging infectious disease threats.

Certainly, this research has certain limitations. Firstly, all in vivo experiments were conducted in IFNAR^−/−^ mouse models. Although this model is highly susceptible to flaviviruses and is a commonly used model for evaluating the safety and efficacy of flavivirus vaccine candidates, its type I interferon signalling pathway deficiency means that it cannot fully simulate the complete immune response and vaccine-induced immune pressure in immunocompetent hosts. Therefore, the observed excellent safety and immune protection effects still require further verification in immunocompetent hosts (such as wild-type mice or non-human primates). Future research will focus on assessing the immunogenicity, protective efficacy, and potential immune mechanism differences in CYV-JEV in models with complete innate immune responses, which will provide more comprehensive experimental evidence for preclinical translation. Secondly, this vaccine candidate primarily targets the JEV GI type, but current safety and efficacy evaluation is still based on the GIII type vaccine strain SA 14-14-2. Although SA14-14-2 can effectively simulate lethal infection in this model, and its E protein is highly homologous with GI wild-type strains, suggesting the possibility of cross-protection, direct challenge experiments against circulating GI wild-type strains are undoubtedly the gold standard for assessing the broad-spectrum protective capacity of the CYV-JEV vaccine. This will be the focus of our next research phase to further confirm its immunological protective advantages for the target genotype. Finally, the long-term immunological persistence of the vaccine needs to be confirmed in subsequent research. This research primarily evaluated the antibody response and protective efficacy in the short term after immunisation. The durability of vaccine-induced immune memory is a crucial factor determining its long-term protective effect. Therefore, systematically assessing the long-term humoral and cellular immune dynamics following CYV-JEV vaccination will be a critical step in advancing the translational application of this candidate vaccine.

## 5. Conclusions

In summary, this research successfully constructed a chimeric vaccine candidate strain CYV-JEV targeting the emerging GI genotype of JEV. Through systematic safety and efficacy evaluation, the results demonstrated that CYV-JEV possesses a certain degree of safety and effective immune protection, successfully transforming the advantages of the ISFV platform into value for actual vaccines. It not only provides a promising candidate vaccine for addressing the challenges brought by JEV genotype transitions but also validates the feasibility of ISFV as a next-generation flavivirus vaccine development platform. Future research directions include verifying its safety and efficacy in immunocompetent animal models, further assessing its protective range, and providing a robust means for the prevention and control of Japanese encephalitis virus.

## Data Availability

All the data in this study were completely preserved in our laboratory. If necessary, they can be obtained by con-tacting the corresponding author.

## Supplementary Materials

The following supporting information can be downloaded at https://www.mdpi.com/article/10.3390/vaccines14010030/s1. Supplementary Material S1: Chimeric virus CYV-JEV nucleic acid sequence; Supplementary Material S2: Chimeric Virus CYV-JEV Construction—Segmented Amplification Products by Agarose Gel Electrophoresis; Figure S1: Flow cytometry gating strategy for peripheral blood; Figure S2: Splenic flow cytometry gating strategy; Figure S3: Chart of Clinical Signs in Mice; Figure S4: Proportion of Paralyzed Mice Chart; Figure S5: Analysis of B Cell Subsets and BCR Characteristics; Figure S6: Analysis of T Cell Subsets and BCR Characteristics.
