# Supplementary material for: Construction and Evaluation of a Chimeric Japanese Encephalitis Virus Vaccine Candidate Strain with Chaoyang Virus as the Backbone
Source: Vaccines (Basel). 2025 Dec 26;14(1):30. doi: 10.3390/vaccines14010030 (PMC12846307; doi:10.3390/vaccines14010030)

Supplementary Material

Supplementary Material S1：**Chimeric virus CYV-JEV nucleic acid sequence**.

CYV-JEV chimeric virus genome sequence, wherein the blue portion represents the GI JEV prME gene sequence：

GAAAAGTATATTCTACGTGTGCATTCTGTAACATAGTTGGTTGGAGAAGTATTTTGGATATAACAC
GTTTGAATAGGTTTATTGTGAATCGAAATACCGATGGCGAATAAACCCAAGAAGCCCGGAAGAC
GGGCTATCGATATAGTGAGACGTGCGTTGCCTCGCGTCTCGGGACCGAAAAAGGTCTTAAAGAA
GGCAACACAAACAGTCATGGAAAGCTTGGCTGGCATACGGGCAACCGTTGCCTACCTGCTCTAC
ATGACATTCCTCGGCAACAAGGTCAGCAAGGCCACGAGAGCTAAGTTTCGCAGTGCGAAAAAG
AGTGATCTGATAAAGATCCTCAGTAGCTTCAAGAGAACGGTTACAAACCTTCTGGCTAGTGTGC
AAAAGAGGAAGAGAAAGGCGAAGAGATCGGTCACGACGCCCAGCATGGCTTTGATTCTATTGA
CAATGAGTTGTATGGCTTATGGAATGAAGCTATCAAACTTTCAAGGAAAGCTTCTGATGACCATC
AACAACACGGACATTGCGGACGTCATCGTGATCCCCACCTCAAAAGGTGAAAACAGATGTTGG
GTCCGAGCAATCGACGTTGGTTACATGTGTGAAGACACCATCACGTACGAATGTCCGAAGCTGG
CAGTGGGCAACGACCCAGAAGACGTGGATTGCTGGTGCGACAATCAAGAAGTCTACGTGCAGT
ATGGTCGCTGCACACGCACCAGGCATTCCAAACGAAGCAGAAGATCCGTATCAGTCCAAACGC
ATGGGGAAAGCTCACTAGTGAACAAAAAAGAGGCTTGGCTGGATTCAACGAAGGCCACGCGAT
ATCTCATGAAAACGGAGAATTGGATCATAAGGAACCCTGGGTATGCTTTCCTGGCGGCGGCACTT
GGTTGGATGCTTGGCAGTAATAGTGGTCAACGTGTGGTGTTTACCATCCTCCTGCTGTTGGTCGCT
CCGGCTTACAGTTTTAACTGTCTGGGAATGGGGAATCGAGATTTCATAGAAGGAGCCAGTGGAG
CCACCTGGGTGGATCTGGTATTAGAAGGAGACAGTTGTTTGACAATCATGGCAAACGACAAACC
AACACTAGATGTCCGCATGATCAACATTGAAGCTAGCCAACTTGCTGAAGTCAGGAGCTATTGCT
ATCACGCTTCAGTTACTGACATTTCAACGGTGGCTCGATGCCCCACGACTGGAGAAGCACACAA
CGAGAAACGTGCTGACAGCAGCTACGTGTGCAAGCAAGGCTTTACTGACCGCGGATGGGGAAA
TGGATGTGGACTCTTCGGGAAAGGAAGCATTGACACTTGCGCAAAATTTTCTTGTACCAGTAAG
GCCATTGGAAGAACAATCCAACCAGAGAACATCAAGTATGAGGTTGGTGTATTCGTGCACGGAA
CCACCACCTCGGAAAACCATGGGAATTACTCAGCGCAGGTCGGAGCATCTCAAGCAGCAAAGT
TTACTGTAACTCCAAACGCTCCTTCAATAACCCTCAAGCTTGGTGATTATGGAGAAGTCACACTG
GATTGTGAACCAAGGAGTGGACTGAATACTGAAGCGTTCTATGTCATGACTGTGGGTTCGAAGT
CATTCTTAGTCCATAGGGAATGGTTCCATGACCTTTCTCTTCCCTGGACGTCCCCCTCAAGCACGG
CATGGAGGAATAGAGAACTCCTCATGGAATTTGAAGAGGCACATGCCACAAAACAATCTGTCGT
AGCCCTTGGGTCACAGGAGGGAGGCCTCCATCAAGCGTTAGCAGGAGCCATCGTGGTGGAGTA
CTCAAGCTCAGTGAAGTTGACATCAGGTCACCTGAAATGCAGGCTGAAAATGGACAAACTGGC
TCTGAAGGGCACAACTTATGGCATGTGCACAGAAAAATTCTCGTTCGCGAAAAATCCAGCGGAC
ACAGGCCATGGAACAGTTGTCATTGAGCTCACATACTCTGGAAGTGATGGTCCCTGCAAAATTC
CGATTGTTTCAGTCGCGAGTTTAAACGACATGACCCCTGTGGGAAGGCTGGTAACAGTAAACCC
CTTCGTCGCGACATCCAGCTCCAACTCGAAGGTGCTGGTTGAGATGGAACCTCCTTTCGGAGAC
TCTTACATCGTGGTTGGAAGAGGGGACAAGCAGATTAACCATCACTGGTACAAAGCTGGAAGCA
CGTTGGGAAAAGCCTTCTCAACAACTTTGAAAGGAGCTCAAAGACTGGCAGCGCTAGGTGACA
CAGCCTGGGACTTTGGTTCCATTGGGGGGGTATTCAACTCCATAGGGAAAGCTGTTCACCAAGTA
TTTGGCGGTGCATTTAGAACGCTCTTTGGGGGAATGTCTTGGATCACGCAAGGACTAATGGGGGC
CTTACTTCTCTGGATGGGTGTCAACGCACGAGACCGGTCAATCGCCCTGGCTTTTTTGGCCACGG

GAGGTGTGCTCGTGTTTTTAGCGACTAATGTGCATGCTGAAATTGGATGCAGCCTTGACATAACA
CACAGAGAAATCAAGTGTGGAGATGGCATTTTCATCTTCAGGGATGCAGGGGGCTGGAGAGAC
AAATATGTCTTTCATCCTGGCTCTCCAAAAACCCTAGCTGCAGCTATTTGGAAAGGATGGAATGA
TGGAATCTGTGGAGTTAGGTCAGCCACCCGCATGGAACATGAAATGTGGAAACAAATTGAAAAT
GAGCTCAATGGGATTCTAGAAGAAAATGACATCAAATTGAGTGTCGTTGTGAAGAATGCCAATG
GCACATATCCACGAGGAACCAAGTCATTAACCCGAAACACAACAGGACTGCAGTACGGATGGA
AGTCGTGGGGCAAGACAATGTTTGTTTCAGTGCCAATAGCCGAGAACACTTTCATAATCGATGG
AAATGATGAAGGAGAGTGTGCCTCGGACAAACGCGCTTGGAATACATTTAAGATAGAGGAGTTT
GGCACAGGAATAATGAAAACCAAAGTCTTCCTGGATTTGGCTGACGCACAAACAGAATACTGC
GACACGGAACTGTTGGGAGCTGCTGTGAAAGGCAACAAGTCTGTTCATGGCGACCCCGGATTGT
GGATGACTGCTTCTAAGGAATCAGGCGACTGGAAACTGGAATCATTGTCAATGACGGAGAGCA
GGCGGTGCCTGTGGCCCGATTCGCACACCATTTGGGGAAGAGGAGTGCTGGAGTCAAAACTCAT
CTTACCATCAATGTTCGGAGGACCAGTTTCTCACATGAACACCAGACCTGGATATGCCACGCAAT
TGTCAGGACCCTGGAACAATGTCCCACTGGATGTTGTGTTTGAGGAGTGTCCTGGTACTAAAGTT
GTGGTGGAAAACAATTGCACGAACAGAGGAGAATCCATCAGATCTACCACAGATAGTGGCAAA
ATCATTCCAGAATGGTGCTGCCGGAAATGCACCATGCCACCCCTCACTTACCGCACGCCAGATG
GATGCTGGTATGCCATGGAAATCAGACCAAAAAAGGCAAGCGAAGAAAGTCTGTTGCGATCAA
AGGTTTCAGCTGGAACATTTCAAGGAATTGATGATTTTTCCTTAGGGCTCCTAGTGCTAATCATTT
TCGTGCAGGAAGGTCTTAAAAGAAGAATGACCAGCCGGTACATAATGTTGGCTGCACTAGGACT
ATTGCTGGCAGCAGTGTTGGGTGACCTCACATACAATGACATAGCAAGGTACGTGATCATGGTTG
GTGTAGCATTTGCAGAAATGAACAACGGGGGAGACTTGATTCATTTAGCCCTGATTGCCACATTC
AAAGTGCAACCTGGTTATCTGCTTTTCTTCCTTCTTCGCAAACAATGGTCACCACGGGAAAGTAC
CATTTTGGCTTCAGCAGCAGTCGTTCTGCAAATTTGTGCGGCTGCATGGCAATCAACAAAGTCTA
TGCAAGTGCTGAATGCTCTTGCTATGGGATGGCTGTACATCAGAGCCATTGTGGTACCCGGAGCT
CTTTCCAAAGCAATGCCCCTCATTTGCATGTGCGTCCCAGGCGTGTTGAGCCTGACGCCACATGC
TATAAGAGTCAGCATGGTTACAATAGCGGCAGGCACTCTCATCAAAGGCACGAAGGGAACATCA
GTTCGGAAGCACATGCCCTATTTTTTAGGACTAGTAGGAGCCGTGGCCGGACTGGATCCATTAGG
TATGCTTGGATACTCCTTATTAACGTACAGCAGCGGAAAGAGGTCATGGCCAGCAGGAGAGATT
ATGACGGCAGTCGGTCTAACATGCGCTATGATCGGGGCATTAAGCGGAAACGCGATGAATGATA
TTGCTGGGCCTGCGGCAGCTGCCTCTCTGATTTTTGTGGCTTACGCAATAAGTGGGAGATCTGCA
GACGTGTTCTTGGAAAAGGCTGGTGAAATTTCATGGATTGACGACGCAGCAGTTTCTGGATCAA
GTCCAAGGGTGGACGTGCAAGTTACCGACGGGGGGGATTTTCGGCTTCGCCACGAAGCCGAGG
CTTCGTGGCTAAAGAATGGAGTGATGGCTTTCTGTCTCGTTCTGGCTGGAGTACACCCGTTAGCG
ATTCCGGTAGCGGGGTTGATCTGGTTCGGTTTCGTAAAAAGTGGGAGGCGTGGCACGGTTCTCTG
GGATATACCACATCCGATTGCATCATCTGCCCCAAGTGTGGAGGACGGATGTTACAGAGTGATGT
CAAGAAGGCTCATTGGTTCTACGCAAGTAGGAGTGGGAGTGATGAAAGACTCAGTGTTTCACAC
AATGTGGCACGTCACCCGTGGCGCCTCTCTAACGAGTGGTAACGGTAGAATGGATCCATATTGGG
CGGATGTTCGAGAAGATCTCATCAGCTACGGTGGGCCATGGAAGCTCAACAGCACTTGGGATGG
ATCTTCTGAAGTGCAGTTAATAGCAGTGCAACCCCAGAAAATACCCGAGAACGTTCAAACAACT
CCAGGAAGGTTCGTCATGTCAGACGGAACTGAGGTTGGGGCTGTGGTGCTTGACTACCCGTCAG
GCACTTCAGGTTCCCCAATCGTGGACAAAGATGGAAATGTGGTTGGACTGTATGGGAATGGTGT
CATGCTTAACGACAGCACCTACGCTAGTGCGATCGCGCAGTCAGCAGGTTGTGAGAACATTACC
CCTGTAGTATTCCACCCGGACATGTTGAAAAAGGGAAAGCTGAGCGTTATGGACCTACATCCAG
GAGCGGGAAAAACCCGCAAGGTGTTGCCACAGGTTCTCAAAGAAGCAGTGGCCAAGAAGTTG

AAAACCCTTGTACTGGCCCCGACGCGTGTTGTAGCTAAGGAAATGCACGGAGCTCTAGCTGGAT
TGCCGGTGAGATACCAAACCTCGGCCGTTGAAACCAAAGGGAGTGGAAATGAGCTGATAGACG
TCATGTGTCATGCGACATTTACGTACAGACAATTGACACCAGGAAGAATGGTTAATTACCAATTG
TATGTAATGGATGAGGCACATTTCACTGACCCAGCTTCAATTGCGGCAAGAGGAATAATCGCAA
CACGGGTCAAGCTAGGAGAAGCAGCTGCGATTTTCATGACGGCAACACCTCCAGGAACTCTTGA
TGCATTCCCAGAATCCAATAGCCATATTGAAGATGAGGAAAGGGAAATTCCAGACAAGGCATGG
AGCACCGGATTCGAATGGATTACAGATTACACAGGCAAAACTGTGTGGTTTGTTCCATCAATTCG
AACTGGGAACATCATAGCGAGCTGCCTCGCGAGGACAGGGAAGAAATGCGTTGTGTTGAATAG
CAAAACATTCAATGACGAGTTTCCAAAGACCAAGAGTGGAACGTGGGACTTTGTGATCACAAC
GGACATATCTGAAATGGGAGCAAATTTCAAAGCCTCACGAGTCATTGACTGTAGAACGTCAATC
AAACCAACATTGGCCTATGTCCCTTCTGAGAGAGTAGTGTTGGGATCTCCAAAACCCATAAGCC
CAGCTAGTGCAGCACAGCGTAGAGGGAGAGTGGGTCGAGACCCCACTCAGCTTGGTGACCAGT
ACATTTACGGAGGCGAGGTTGGTGACGACTTCTCCAACATGGTGCACTGGACAGAAGCGAAGA
TCTTAATGGACAACATCATGGTACCAGGCGGTCTGTACCCACAGTTCTACGAGCCGGAGGCTGA
CATGCTAAGTGAAACGGATGGACACTTCAGGCTGGATATGACCAAACGTGACGTATTCAAGGAT
TTGGTGCGCAAGGCAGACTTACCCATATGGCTGGCTTATCAGGTGGCCAAGCATGGTCATGAATA
CAAAGACAGAAGCTGGTGCCATAGTGGGCCATCTGGACATCTCATCTACGATGATTACGGACAG
ACAGTTGAATACAAGCTTGTGAATGGCGAGAGGAAGATACTGCAACCAAGATGGATTGACCAA
CGCACATACCAGGAGAAAACAGCACTCAAGGCTTTCATCGAATTTGCTGAGGGACGCCGAAGTT
ATGTTCCTATAATAGAAGTGCTCGGCAAACTTCCACAGCATTTCGCCGACAAAACTATTGATGCG
GCTGACACCTTCAAGACTGTGCTTACAGCCACTCCAGGAAGTAGAGCATACAGGCTTGCAGTTG
ACAACCTACCCGATGCAGCAGAGACGGCAATATTCGTGACGATGGTAGGGTTCATGACCATGGG
AATACTAATTTTCCTGATGGCACCCAAAGGCATGACACGCATGTCGCTGGGATTCATGACCATCA
TGGCAGCTACGTATTTTCTGTGGGCTTCAGGCATGGCTGGATATCAAATAGCGGCAATGCAATTA
GTGGCTTTCATTCTCTTCGTAGTCTTGGTTCCAGAACCAGGATCGCAGAGATCAGTACAAGACAA
TACGATTGCCATGGTCCTAATTGGAATACTAAGCATTGCAGCATTGATTGCGGCAAATGAGACAG
GACTGCTGGAGAAGACAAAAAATGATTTCGTGTCTTTGTTACCGGTTAAAGCCCCGAACAACAA
TCCCTGGAAGTTTGATTTTAGCGTGGACTTGAGACCGGCGACATCATGGGCTTTGTATGTAGTGAT
TGCTACAATGCTTGGGCCCATTTTGGAGCATGCGATTGTGACTCACTTCACAAGTATATCAATTGC
AGCCATCACGAATCAAGCAGGAATACTGCTATCAATGGATAAAGGAACGCCATTTTTCAACTTG
GACTGGGGCGTCATTCTGTTGGGAGTGGGGTGTTGGTCTAGCATAACGGGGACAACCTTGTTGTG
TGCGATCGGACTCTCCTGCGTTCACTTCTCAATGACTCTACCTGGAGTCAGGGCTAAAGCAGCGA
GAGAAGCTCAAAACAGAACCGCAGCTGGAGTATCAAAGAACCCTATTGTGGATGGAGTCAACA
CTATAAACGTCACAGCGCCCCCAGGAATGGACCCCATGTATGAGCGCAAACTTGGACTGTGGAT
GCTCCTGATCATGGCGGTCATATCCACAGCGGTAAACAGAGACTTGACTCACTTGATGGAACTA
GGCATACTGGGAAGCGCAGCACTTGGCCCACTCATAGAAGGGAACTCTTCCACAATCTGGAACA
CCTCAGTGGCATCTTCACTGTGCAACTTGATGAGAGGACAGTATCTAGCTGGAATACCTCTGACA
TACACGCTGGTCAGAAACCTCTCGCTAAAAGGGGTTCCCCGTAGAGGAGTCACCATAAGCAAC
ACCCGAGGGATGGAATGGAAACGCAAACTAAACGCAATGAACAAGGAAACCTTTCAGAGATAC
AGACGAGATGGGATAGTTGAGGTGGACAGAACAGCAGCGCGGGAAGCTTTAAAGAGTGGAAA
TGTAACAGGAGGTCACGCTGTATCCCGGGGTTCAGCAAAGCTCAGATGGCTGGTAGATAAAGGA
TATTTACGCCTGATGGGGGATGTCACAGACTTGGGATGCGGGCGAGGTGGATGGTGTTATTACGC
AGCAGCTCAAAGAAACGTTGTATCTGTACGAGGCTTCACCAAAGGAGGTGACGGACACGAAGA
ACCCATTCCGGTGCAGACTTATGGATGGAACATTGTCACTCTGCGTAGCAAAGTGGACGTTTTCT

ACATGCCTACACACATGACAGACACTCTATTGTGTGACATTGGAGAGTCATCGTCTAACATGCTA
GTAGAAGAAGAGAGGACCCTACGCGTGCTGAATCTGGTTGAAGAGTGGATCAGAAAGAGTAAG
CCAACTCACTTTTGTTGCAAGGTTCTTGCTCCATACATGCCAAATGTATTGGAGAAGATAGACAA
GCTAATGAAAGTTCATGGAGGTGCTTTAGTGAGAGTGCCACTCTCCCGCAACAGCACACATGAG
ATGTATTGGGTATCTGATGCAAGAGGTAATGCCATGAATGCAGTGGCATCTCTGAGTAGAACACT
TTTGGACAGGATGATACACTTGACAGGAAAAGTGCACTGGGAAGATGATGTCAACCTGGGAACT
GGGACTAGAGCAGTGAACAGTGTCGCTGATCCACCAAACTGGGACAAAATTGGACTCCGAGTG
AGAAAACTTGAACAAGAATACAAAGCTTCATGGATGTATGATGCAGAACACCCCTACAAGACG
TGGACCTACCATGGATCATATGAGACTTCCACATCTGGAAGTGCTTCATCAATGATAAATGGAGT
GGTCAAGGAGCTAACACACCCATGGGACACAAACTCCGGAGTGACAAACGTTTGCATGACAGA
CACAACCCCTTTTGGGCAACAGCGCGTTTTCAAAGAAAAGGTCGACACCAAAGCAATGGAACC
TCCAACTGGAACCAGAGAAGTGATGAGGATTGTGAACAGATGGTTGACAAACTACCTGTCTAGA
ACAAAGAAGCCTAGACTGTGTACTCCAGATGAGTTTATTGCTAAGGTGAACTCTGATGCGGCTCT
GGGAACAATGTTCAATGACCAAGGGAACTGGCCAAGCGCAAAAGATGCAGTGAGAGACCATG
GATTCTGGAGGCAAGTCGACATTGAGCGACAACACCACTTAGAGGGAAGATGCGTCTCATGTGT
CTACAACATGATGGGAAAGCGTGAAAAGAAATTGACGGAATTTGGAAGGGCCAAGGGCAGCA
GAGCAATCTGGTACATGTGGCTAGGAGCAAGGTACTTGGAATTTGAAGCCCTAGGATTCCTGAA
CGAAGATCATTGGCTCTCGAGAGAGAATTCCAAAGGTGGGGTCGAAGGGATTGGATTACAATAC
CTAGGCTACATCCTTGAGGATATGGGAGGAATGACAGGAGGCCAAATGTATGCAGATGACACAG
CCGGCTGGGACACCAAGATAACGAATGCTGACCTAGAGGATGAGATGGAGATCACAAAGCTGA
TGGCACACACCCACAAAAAATTGGCCACGGCCATTATGGATCTCACGTACATGAACAAGGTTGT
GAGAGTGATGAGGCCAGGCAAGGGAGGAAAGACGCTAATGGACATCATTAGCAGGAAGGACC
AGAGAGGTAGCGGACAGGTCGTGACTTACCCCCTCAACACGTGGACAAACCTCAAGGTGCAGT
TGATTAGGATGGCTGAAGGCGAAGGAGTGATCCTACCTGAAGACACTCTGTCACTCACCGGCTG
CAGTAGACGCAATTTAGAAATGTGGCTGGTGAGGAACGGAGAAGAACGATTAACCCGGATCGC
TGCTAGCGGAGATGATGTGGTGGTTAAGCCCATTGATGACCGATTTGCAGAAGCTCTGCACTTTC
TCAACAGCATGGCCAAGATCAGAAAAGATATCAATGAATGGAAGCCCTCAACAGGCTGGAACA
GTTGGGAGGGTGTGCCGTTTTGTTCACACCATTTCCACAAGCTCAGCTTAAAGGATGGAAGAAC
ACTCACAGTTCCATGCAGAGATCAGGACGAACTTATTGGAAGAGCTAGAGTGTCTCCTGGAGCA
GGGTGGACGTTGCGAGAAACCGCAGGACTCAGTAAGGCGTATGCGCAAATGTGGATGCTGATCC
ACTTCCACAGAAGGGATCTACGAACCATCGCGTTCGCAATTTGCAGTGCAGTGCCTAAAGACTG
GGTTCCCACAGGAAGGACATCGTGGTCCATCCATGCTCGAGGTGAATGGATGACGAATGAAGAC
ATGTTGGCGGTGTGGAATCGAGTGTGGATCACTGAGAATCCATACATGTTCAACAAAGAAACAA
TTCACGACTGGCGAGATGTTCCATACCTGCGGAAACAACTGGACAAAAATTGCGGATCAATGAT
AGGAGTGAGGTCCAGAGCCACGTGGGCTGAAAACATTCGTGTTTCAGTGAACCAGGTACGTGG
TTACATTGGTAAACATGAGAACTACCTCGATTACCTCCAAGCCCAGAATAGATTCAGCATTCCTG
CCGAATTCACCATGGGCAACATATTGATCTAAGACACTGAGAGAAAAGACTGAAAAACAATTG
ACGCTGAGAGTCAGGCCTAAATGCCACCGGATGATAGTAGACGGTGCTGCCTGCAGCTATCACA
TATAAACTGGCGCCTTATATGTTTATTAGCCGACAGGGGGACACATGCACCCAGCAGCCCGAGCT
GGACAAGGCATGTGTACTAGCGGTTAGAGGAGACCCCCCCAAAAATGAAGGGCACTATATCGA
CACTTGGGAAAGACCAGAGGTACTCGCTGATTCACCGCCACCAGACTACACGGCACAGCGCGC
CGGAAAAGGTAGTTTGGAGGTTGTAAAACAACAAGTATCT

Supplementary Material S2：**Chimeric Virus CYV-JEV Construction - Segmented Amplification Products by Agarose Gel Electrophoresis.**

Six DNA Fragments Amplification Primers and Amplification Product Identification：

Primer name and sequence

**Primer name**	**Sequence（5′-3′）**
CYV-JEV-1F	CTATAAATACAGCCCGCAACGATCTGGTAAACGAAAAGTATATTCTACGTGTGC
CYV-JEV-1R	GGTCATCAGAAGCTTTCCTTGAAAGTTTGATAGCTTCATTCCATAAGCCATACAACTCATTGTCAATA
CYV-JEV-2F	TTTGATTCTATTGACAATGAGTTGTATGGCTTATGGAATGAAGCTATCAAACTTTCAAGGAAAGCTTCTGATG
CYV-JEV-2R	GTGTTATGTCAAGGCTGCATCCAATTTCAGCATGCACATTAGTCG
CYV-JEV-3F	TTAGCGACTAATGTGCATGCTGAAATTGGATGCAGCCTTGACATAA
CYV-JEV-3R	TTTCAACATGTCCGGGTGGAATAC
CYV-JEV-4F	GTGTCATGCTTAACGACAGCACCT
CYV-JEV-4R	CGCTCATACATGGGGTCCAT
CYV-JEV-5F	GAACCCTATTGTGGATGGAGTCAACA
CYV-JEV-5R	GGAGGCTGGGACCATGCCGGCCAGATACTTGTTGTTTTACAACCTCCAAACT
CYV-JEV-6F	CACCAGACTACACGGCACA
CYV-JEV-6R	ACCAACTATGTTACAGAATGCAC

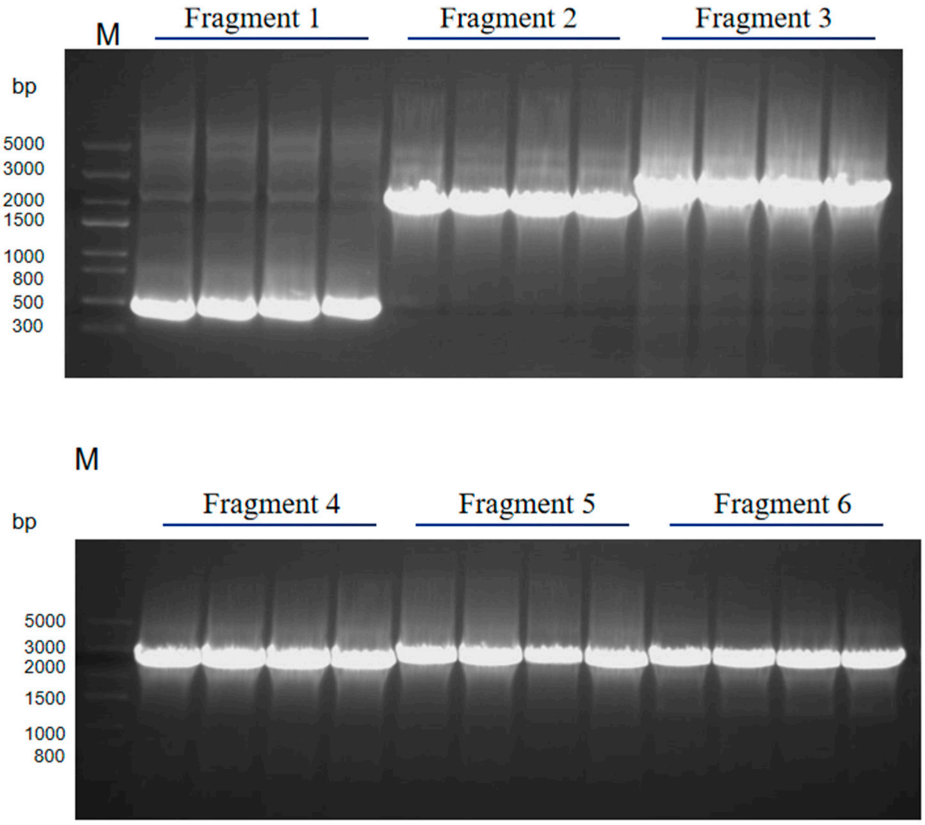
M
Fragment 1
Fragment 2
Fragment 3
bp
5000
3000
2000
1500
1000
800
500
300
M
Fragment 4
Fragment 5
Fragment 6
bp
5000
3000
2000
1500
1000
800

Figure S1: **Flow cytometry gating strategy for peripheral blood**

CD19⁺ to gate B cells, CD3⁺ to gate T cells, then further gating CD4⁺ T cells and CD8a⁺ T cells (red). From CD19⁻CD3⁻ cells, gate CD11b⁺Gr1⁺ neutrophils (yellow) and CD11b⁺F4/80⁺ macrophages (yellow).

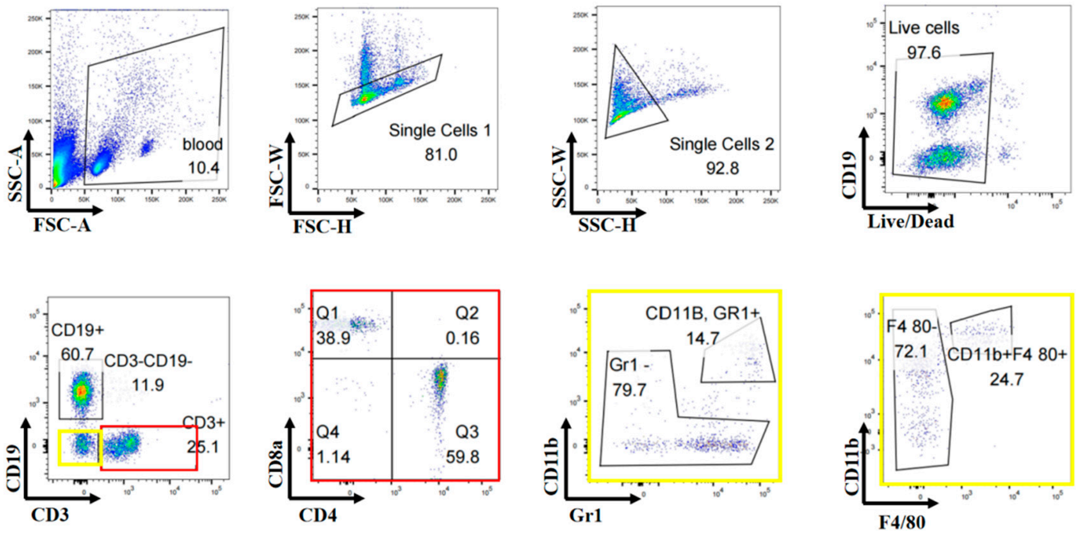

Figure S2: **Splenic flow cytometry gating strategy**

$CD19^+$ gated for B cells, $CD3^+$ gated for T cells, further gating of $CD4^+$ T cells and $CD8a^+$ T cells (in red). From $CD19^-CD^{3-}$ cells, gating of granulocytes and macrophages (in yellow).

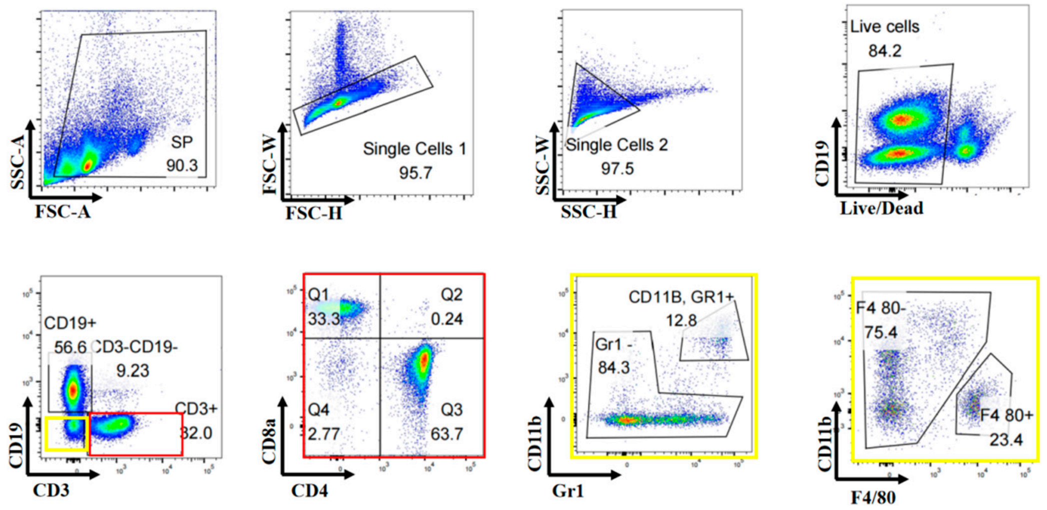

Figure S3: **Chart of Clinical Signs in Mice**

All JEV-SA-infected mice exhibited typical neurological symptoms, including arched back, ruffled fur, lethargy, and limb paralysis, whereas mice in the CYV-JEV group showed no abnormal clinical signs.

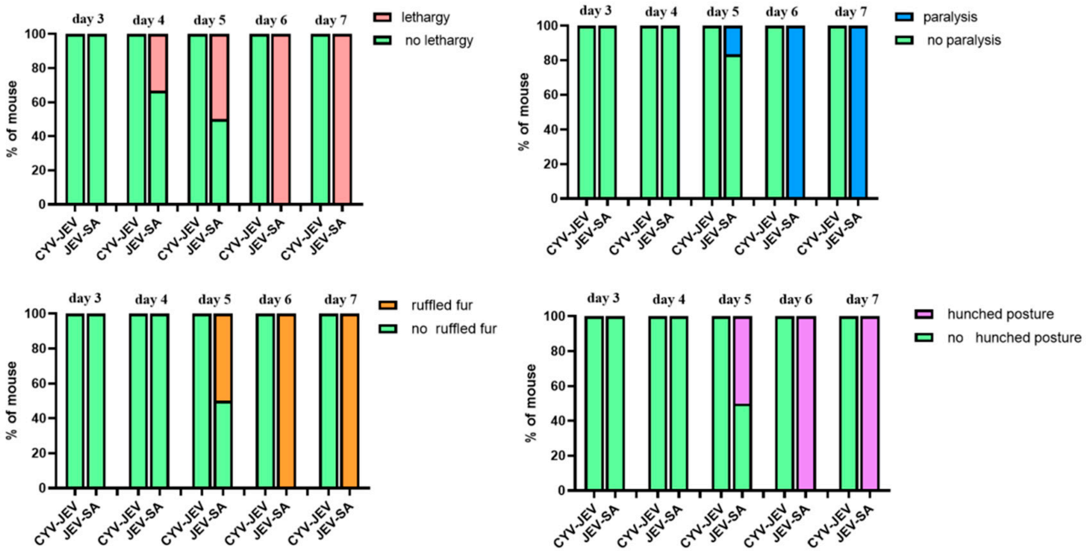

Figure S4: **Proportion of Paralyzed Mice Chart**

Proportion of Paralysed Mice Following JEV-SA Challenge with Different CYV-JEV Immunisation Dosages and Immunisation Frequencies

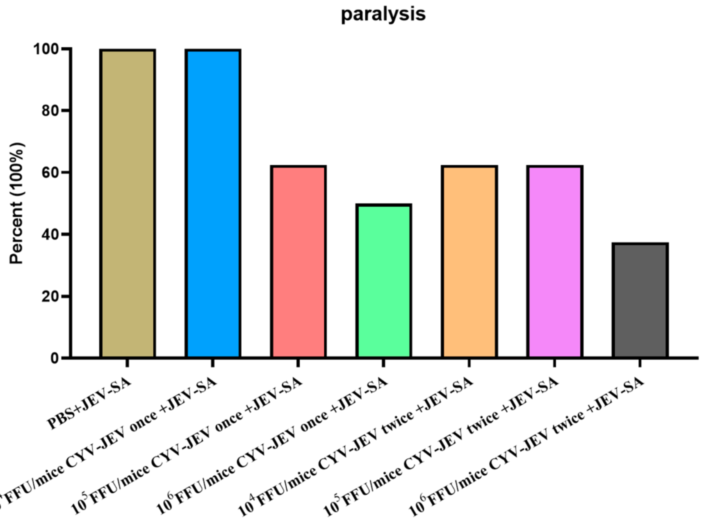

Figure S5: **Analysis of B Cell Subsets and BCR Characteristics**

B Cell Subpopulation Clustering Annotation Results and BCR Composition Changes in Memory B Cell Subpopulations

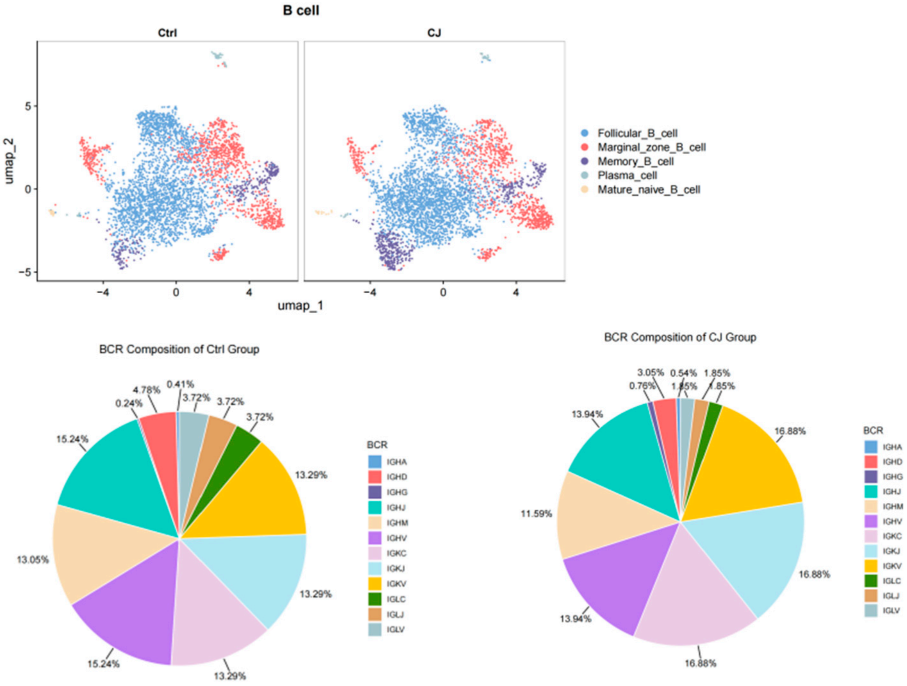

Figure S6: **Analysis of T Cell Subsets and BCR Characteristics**

T Cell Subpopulation Clustering Annotation Results and TCR Composition Changes in Effector CD8+ T Subpopulations

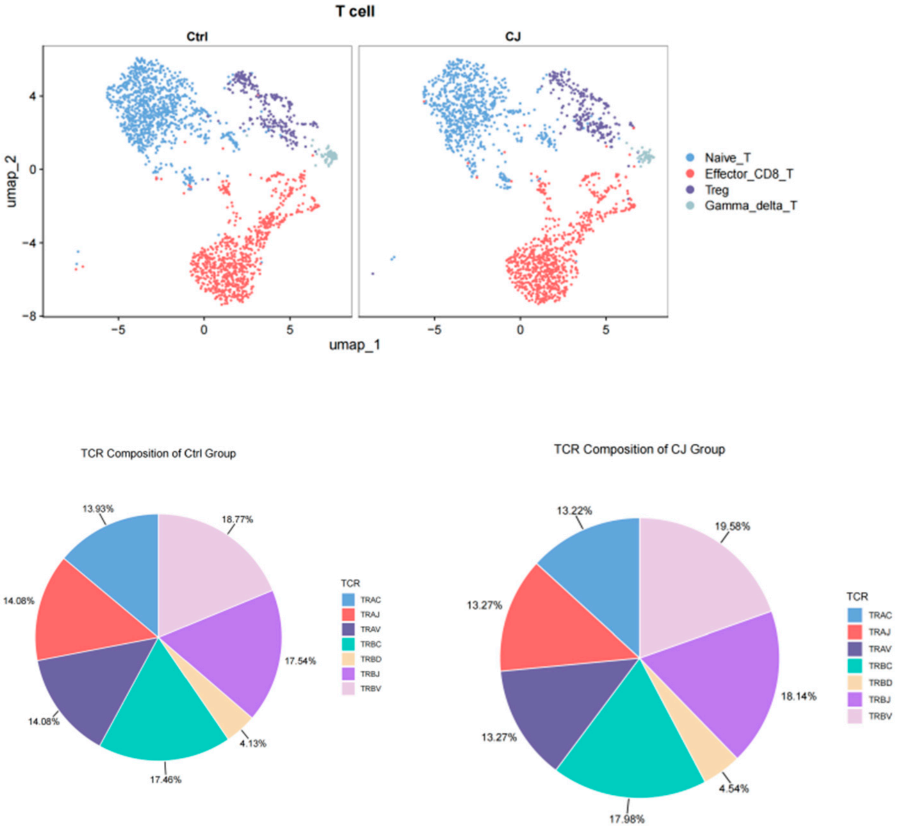

Supplement: Supplementary file 1 [file vaccines-14-00030-s001.zip › vaccines-4060128-supplementary.pdf]
